# Importance of Functional Loss of FUS in FTLD/ALS

**DOI:** 10.3389/fmolb.2018.00044

**Published:** 2018-05-03

**Authors:** Shinsuke Ishigaki, Gen Sobue

**Affiliations:** ^1^Department of Neurology, Nagoya University Graduate School of Medicine, Nagoya, Japan; ^2^Department of Therapeutics for Intractable Neurological Disorders, Nagoya University Graduate School of Medicine, Nagoya, Japan; ^3^Brain and Mind Research Center, Nagoya University, Nagoya, Japan; ^4^Research Division of Dementia and Neurodegenerative Disease, Nagoya University Graduate School of Medicine, Nagoya, Japan

**Keywords:** FUS, FTLD/ALS, tau, GluA1, SynGAP

## Abstract

Fused in sarcoma (FUS) is an RNA binding protein that regulates RNA metabolism including alternative splicing, transcription, and RNA transportation. FUS is genetically and pathologically involved in frontotemporal lobar degeneration (FTLD)/amyotrophic lateral sclerosis (ALS). Multiple lines of evidence across diverse models suggest that functional loss of FUS can lead to neuronal dysfunction and/or neuronal cell death. Loss of FUS in the nucleus can impair alternative splicing and/or transcription, whereas dysfunction of FUS in the cytoplasm, especially in the dendritic spines of neurons, can cause mRNA destabilization. Alternative splicing of the *MAPT* gene at exon 10, which generates 4-repeat Tau (4R-Tau) and 3-repeat Tau (3R-Tau), is one of the most impactful targets regulated by FUS. Additionally, loss of FUS function can affect dendritic spine maturations by destabilizing mRNAs such as Glutamate receptor 1 (GluA1), a major AMPA receptor, and Synaptic Ras GTPase-activating protein 1 (SynGAP1). Moreover, FUS is involved in axonal transport and morphological maintenance of neurons. These findings indicate that a biological link between loss of FUS function, Tau isoform alteration, aberrant post-synaptic function, and phenotypic expression might lead to the sequential cascade culminating in FTLD. Thus, to facilitate development of early disease markers and/or therapeutic targets of FTLD/ALS it is critical that the functions of FUS and its downstream pathways are unraveled.

## Introduction

Amyotrophic lateral sclerosis (ALS), characterized by selective motor neuronal loss in the central nervous system, and frontotemporal lobar degeneration (FTLD), which is distinguished by changes in character, abnormal behaviors, language impairments, and progressive dementia, have recently been recognized as two ends of the spectrum of one disease (Robberecht and Philips, 2013). This notion is supported by the genetic determinants underlying familial FTLD/ALS (Renton et al., 2014) and lines of evidence showing a pathological continuity between ALS and FTLD (Riku et al., 2014). RNA binding proteins (RBPs) such as transactive response (TAR) DNA-binding protein 43 (TDP-43), and fused in sarcoma (FUS) genetically and pathologically link the two neurodegenerative diseases to a single disease state (Van Langenhove et al., 2012). These genes are causative for familial ALS and FTLD, and are pathological hallmarks of both familial and sporadic FTLD/ALS in which TDP-43 or FUS-positive inclusions are observed (Kwiatkowski et al., 2009; Lagier-Tourenne and Cleveland, 2009; Vance et al., 2009; Mackenzie et al., 2011; Strong and Volkening, 2011). Additionally, FTLD has also been classified as a tauopathy characterized by an accumulation of phosphorylated microtubule-associated protein tau (Tau) in affected neurons (Seelaar et al., 2011).

FUS was originally identified as a fusion protein that resulted from a chromosomal translocation in human myxiod liposarcomas, in which the N-terminal portion of FUS was translocated and fused to the transcription factor CHOP (Crozat et al., 1993). FUS functions as a regulator of multiple aspects of RNA metabolism, including transcription, alternative splicing, and mRNA transport, as well as DNA damage regulation (Bertolotti et al., 1996; Wang et al., 2008; Schwartz et al., 2012; Tan et al., 2012). Whole-body knockout of FUS in highly homogenous inbred C57B6 strain mice resulted in early neonatal death due to immune system defects (Hicks et al., 2000), whereas FUS KO in outbred mice had no developmental impairments (Kuroda et al., 2000). These suggest that the inbred background lacks genes required to compensate for the FUS KO effects. Similar to TDP-43 pathology, FUS-related FTLD/ALS pathology is characterized by mislocalization of FUS to the cytoplasm and a concomitant reduction in nuclear expression in affected neurons (Neumann et al., 2009; Deng et al., 2010; Mackenzie et al., 2011). Redistribution of FUS from the nucleus to the cytoplasm implies that the loss of nuclear FUS is causal for FUS-associated ALS/FTLD. Indeed, loss of FUS leads to neuronal cell death in *Drosophila* and zebrafish (Kabashi et al., 2011; Wang et al., 2011). On the other hand, accumulation of FUS in the cytoplasm is strongly associated with stress granules, which are non-membranous, cytoplasmic ribonucleoprotein (RNP) granules composed of mRNAs, translation initiation factors, ribosomes, and other RBPs. These granules are induced by various cellular stresses, such as oxidative stress, glucose starvation, mitochondrial dysfunction, and viral infection that inhibit translation initiation. The stress granule associated gain-of–toxicity hypothesis of FUS has been well reviewed elsewhere (Gao et al., 2017).

This review provides an overview of recent findings that reveal the effects of functional loss of FUS on the pathogenesis of FTLD/ALS. First, loss of FUS in the nucleus leads to imbalanced Tau isoforms due to insufficient skipping of exon 10 in the *MAPT* gene. Second, loss of FUS in the cytoplasm causes decreased stability in GluA1 and SynGAPα2 mRNA resulting in aberrant maturation of dendritic spines. In addition, we summarize the roles of FUS in neurite maintenance and axonal transport, and provide a briefly overview of the FUS liquid-phase-transition, which may alter its various physiological functions and contribute to the development of toxic cellular effects under pathological conditions. Thus, the functional properties of FUS may influence multiple cellular processes of neurons and/or glial cells whose dysfunction could be the most plausible explanation for neuronal toxicity mediated by loss of FUS.

## Quantitative and qualitative loss of function of FUS

Although recent reports have suggested that loss-of-FUS-function in motor neurons might not contribute to motor neuron degeneration in ALS (Scekic-Zahirovic et al., 2016; Sharma et al., 2016), lines of evidence suggest that loss-of-FUS-function in cerebral neurons can contribute to neuronal dysfunction and neurodegeneration in FTLD. FUS-deficient mice generated either via silencing or FUS knock-out exhibit behavioral impairments (Kino et al., 2015; Udagawa et al., 2015). However, recovery of wild-type FUS in the FUS-silenced mice rescued the behavioral phenotypes, whereas a disease-associated mutant did not (Ishigaki et al., 2017).

Although FUS pathology is detected in both ALS and FTLD cases, the majority of disease-causing mutations within FUS are associated with ALS cases. Nevertheless, a subset of familial and sporadic ALS cases involving FUS gene mutations have been shown to have cognitive dysfunction or mental retardation (Bäumer et al., 2010; Huang et al., 2010; Yan et al., 2010; Belzil et al., 2012; Yamashita et al., 2012). Moreover, a spectrum of cognitive impairments have been observed in a considerable subpopulation of ALS patients (Swinnen and Robberecht, 2014). Taken together, the clinical data and FUS-silenced mice model findings support the hypothesis that FUS dysfunction results in early cognitive impairments.

**Figure 1:**
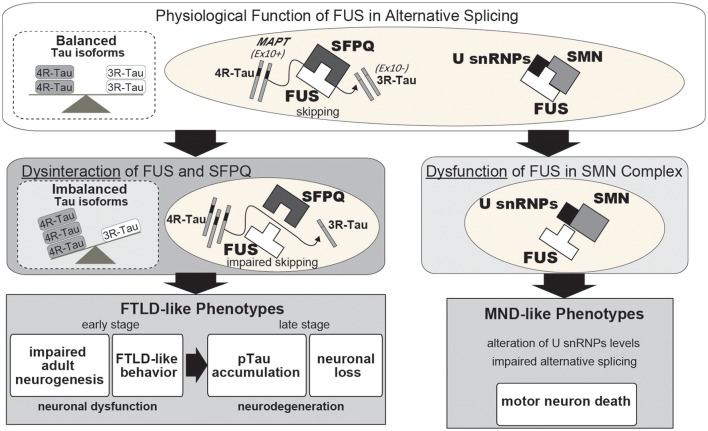
Proposed pathway underlying neurodegeneration following a qualitative loss of function of FUS in alternative splicing. Under normal neuronal physiological conditions, FUS and SFPQ interact in the nucleus to regulate alternative splicing of *MAPT* by skipping exon 10. When this functional machinery is impaired, such as occurs following FUS or SFPQ depletion, or qualitative loss due to disease-associated mutations or other unknown aberrant modifications, the splicing ratio of *MAPT* exon 10+/exon 10- is increased, which in turn results in an increased 4R-Tau/3R-Tau ratio. The quantitative or qualitative loss of FUS or SFPQ causes various phenotypes, including reduced neurite outgrowth, aberrant adult neurogenesis, FTLD-like behavioral impairments, hippocampal atrophy with neuronal loss, and phosphorylated Tau accumulation. Normalization of imbalanced Tau isoforms by co-injection with shRNA against 4R-Tau can successfully rescue these phenotypes. Thus, our findings suggest that a pathophysiological link between FUS/SFPQ and the regulation of 4R-Tau/3R-Tau isoforms is involved in the pathogenesis of FTLD and 4R-tauopathy. FUS also binds to the U-rich small nuclear ribonucleoproteins (U snRNPs) and SMN complexes in the spliceosome with disease-associated mutations in FUS affecting this alternative splicing machinery.

In familial and sporadic FTLD/ALS cases, which are, respectively, characterized by mutations in the FUS coding sequence or the presence of a basophilic inclusion body (BIBD), the affected motor neurons exhibit dislocation of FUS with the protein accumulating in the cytoplasm rather than the nucleus. Cytoplasmic mislocalization of FUS is presumably the first step in the disease cascade; therefore, quantitative loss-of-FUS is thought to be causal for FTLD/ALS. However, disease-associated mutations do not trigger complete mislocalization of FUS to the cytoplasm as a moderate amount of the protein remains localized in the nucleus (Kino et al., 2011). This implies that the FUS mutants are non-functional, and that this, in conjunction with the quantitative reduction in protein, culminates in neuronal dysfunction and FTLD/ALS pathophysiology. It has been reported that FUS binds U-rich small nuclear ribonucleoproteins (snRNPs) and the SMN cmplex, which is the machinery for snRNP biogenesis, and hence compromises precursor mRNA splicing, leading to FUS-associated FTLD/ALS (Tsuiji et al., 2013; Sun et al., 2015). In our recent study, the presence of disease-associated mutations in FUS disrupted formation of a high molecular weight FUS complex by impeding interactions with a second protein, Splicing factor, proline- and glutamine-rich (SFPQ). The impaired FUS functionality suggests that the pathophysiological features of FTLD/ALS also arise from qualitative losses in FUS and SFPQ (Ishigaki et al., 2017; Figure 1). Another group recently reported on the presence of possible SFPQ mutations in familial ALS cases (Thomas-Jinu et al., 2017). These findings suggest that aberrant interactions between FUS and its spliceosome binding partners in the nucleus of neurons might lead to neuronal dysfunction and subsequent neurodegeneration. However, future pathological studies examining the FUS/SFPQ nuclear interaction in both FTLD/ALS and tauopathies are necessary.

The cell selectivity of neurodegenerative diseases such as FTLD/ALS has remained a mystery. For instance, the pathology of FTLD/ALS in both motor neurons and cortical neurons involves major selective neuronal vulnerability. Glial cells, such as astrocytes and microglial cells, have also been linked with FTLD/ALS as modifiers of the non-cell-autonomous mechanism of disease pathogenesis, while cerebellar neurons are typically spared in FTLD/ALS (Boillée et al., 2006; Yamanaka et al., 2008a,b). The cell and region specific-selectivity of FTLD/ALS, however, cannot be explained by the expression pattern of FUS since it is expressed ubiquitously throughout the CNS (Kwiatkowski et al., 2009). We previously found that the profiles of FUS-mediated gene expression and alternative splicing in motor neurons are similar to those of cortical neurons, but differ from those in cerebellar neurons despite the similarity of their innate transcriptome signature. The gene expression profiles in glial cells were similar to those in motor and cortical neurons. Given that motor and cortical neurons are the major affected cell-types in FTLD/ALS, whereas glial cells are modifiers and cerebellar neurons are spared, it is possible that the FUS-regulated transcriptome profiles in each cell-type may determine the cellular fate in association with FTLD/ALS and that neuron-glia interactions may be involved in the pathogenesis (Fujioka et al., 2013). Indeed, FUS silencing caused glial cell proliferation in the brain of non-human primates (Endo et al., 2017).

Taken together, these findings indicate that both quantitative and qualitative losses of FUS function are likely involved in the pathogenesis of FTLD/ALS, and should provide clues for therapeutics that clarify the functional properties of FUS.

## FUS function in the nucleus: regulation of alternative splicing and transcription

Since FUS plays a role in multiple aspects of RNA metabolism, transcriptome deterioration could be the most plausible explanation for neuronal toxicity mediated by loss-of-FUS. In support of this, numerous neuronal function-associated molecules in FUS-regulated transcriptome profiles have been identified (Ishigaki et al., 2012; Lagier-Tourenne et al., 2012; Rogelj et al., 2012; Fujioka et al., 2013; Honda et al., 2013; Nakaya et al., 2013). The alteration of gene expression and/or alternative splicing of these genes may have a large impact on neuronal function which contributes to the neurodegeneration observed in FTLD/ALS. We speculate that disruptions to FUS functionality could result in a partial effect rather than fatal damage by altering isoforms or expression levels of these genes. Thus, it is possible that neurodegeneration only results after the transcriptional disruption triggered by loss of FUS functionality reaches a critical threshold such that the expression of individual genes and alternative splicing events are not critical by themselves.

To gain a better understanding of this mechanism it is necessary to narrow down the list of FUS-regulated genes to those most likely to be disease-associated. Among the genes, alternative splicing of *MAPT* exon 10 has been shown to be relevant to FTLD/ALS pathogenesis (Orozco et al., 2012; Ishigaki et al., 2017). *MAPT* encodes the Tau protein, a microtubule-binding protein in which aberrant accumulation of the phosphorylated form in affected neurons causes tauopathies, such as Alzheimer's disease and FTLD. It has been reported that the ratio of 4-repeat Tau (4R-Tau)/3-repeat Tau (3R-Tau) is high in tauopathies, including FTLD, progressive supranuclear palsy (PSP), and corticobasal degeneration (CBD) (Hong et al., 1998; Yoshida, 2006; Umeda et al., 2013). Our previous study demonstrated that the intranuclear FUS/SFPQ complex regulates alternative splicing of *MAPT* exon 10, which generates two Tau isoforms harboring either three or four microtubule-binding repeats (3R-Tau and 4R-Tau), respectively (Ishigaki et al., 2017). FUS- or SFPQ-silenced mice exhibit an increase in 4R-Tau leading to FTLD-like behavior, reduced adult neurogenesis, phosphorylated Tau accumulation, and neuronal loss (Figure 1; Ishigaki et al., 2017). These findings suggest that the impaired Tau isoform ratio generated in response to dysregulation of alternative splicing by the aberrant FUS-SFPQ complex could be an early pathogenic factor for FTLD/ALS and tauopathies. A report of familial FTLD characterized by a Q140H substitution in FUS with accompanying abnormal Tau isoform ratios supports the idea (Ferrer et al., 2015).

Additional targets of FUS-mediated exon skipping could likewise contribute to FTLD/ALS pathogenesis. Among these genes is FUS itself in which FUS-mediated splicing at exon 7 contributes to autoregulation of expression with the exon 7 skipped variant undergoing nonsense-mediated decay (NMD). The auto regulatory function is deficient in ALS-associated FUS mutants (Zhou et al., 2013).

Other FUS-regulated genes, such as *NTNG1* or *BRAF*, which could be important for neuronal cell survival, have been identified in multiple reports (Orozco and Edbauer, 2013). Further study is necessary to evaluate their significance in FTLD/ALS pathogenesis.

## Function of FUS in the dendritic spine: mRNA stabilization

While FUS is enriched in the nucleus, a percentage of the protein is localized to the soma and neuronal processes (Fujii and Takumi, 2005; Aoki et al., 2012; Yasuda et al., 2013). Moreover, in dendrites many RNA binding proteins, including FUS, are involved in the local translation machinery to regulate synaptic function and morphology (Fujii and Takumi, 2005; Qiu et al., 2014; Sephton et al., 2014). Binding of FUS to the 3′UTR of target mRNAs is an important determinant of translational efficiency and mRNA stability (Colombrita et al., 2012; Lagier-Tourenne et al., 2012; Rogelj et al., 2012). Thus, these findings suggest that the cytoplasmic function of FUS may be involved in regulating mRNA stability, translation, and transport.

Masuda et al. reported that FUS participates in the alternative polyadenylation machinery with FUS binding nascent RNAs and interacting with the CPSF and CSTF complexes (Masuda et al., 2015). In addition, we have shown that FUS regulates GluA1 mRNA stability in cooperation with CPSF6, PAN2, and PABP, while it also controls the mRNA stability of SynGAPα2, an isoform of SynGAP1, with ELAVL proteins in a 3′UTR length-dependent manner. FUS-silencing reduced the number of mature dendritic spines both *in vitro* and *in vivo*. Recovering expression of either GluA1 or the SynGAPα2 isoform in FUS-deficient mice partially ameliorated abnormal behaviors and the impaired dendritic spine maturation caused by FUS-depletion, suggesting that FUS-mediated GluA1 mRNA stability and control of SynGAPα2 isoform-specific expression is critical for these phenotypes (Udagawa et al., 2015; Yokoi et al., 2017).

These results, taken together, suggest that the loss of regulatory control of synaptic molecule mRNA stability in response to impaired FUS functionality causes synaptic dysfunction and could lead to post-synapse impairments in FTLD/ALS.

## Maintenance of neuronal morphology by FUS

It is known that post-synapse impairments in neurodegenerative disorders including FTLD/ALS might be an early pathological change (Sephton and Yu, 2015; Herms and Dorostkar, 2016). For instance, missorting of Tau protein into the somatodendritic compartment is recognized as an early pathological event in Alzheimer disease (AD) and other tauopathies (Ballatore et al., 2007; Hoover et al., 2010). Similarly, FUS^R521G^ transgenic mice exhibited a reduction of dendritic arbors and mature spines (Sephton et al., 2014), and overexpression of FUS^R521C^ exhibited dendritic and synaptic defects accompanied with damaged splicing of *Bdnf* (Qiu et al., 2014).

It was demonstrated that neurite outlength is reduced in FUS-silenced primary cortical neurons but can be recovered by overexpressing wild-type FUS, whereas disease-associated mutants had no effect (Ishigaki et al., 2017). Similarly, iPSC-derived neurons in familial ALS patients harboring mutations in FUS exhibited shorter neurites compared to controls (Ichiyanagi et al., 2016). Moreover, rescue by co-silencing 4R-Tau ameliorated the toxic effects of FUS-silencing on neurite outlength (Ishigaki et al., 2017). Thus, FUS dysfunction induces abnormal neuronal morphology, which may be attributable to alterations in tau isoforms. Indeed, 4R-Tau functions in suppression of microtubule dynamics by stabilizing microtubule interactions and 4R-Tau overexpression affected neurite outlength in a dose-dependent manner (Panda et al., 2003; Ishigaki et al., 2017). Thus, the morphological abnormalities in neurites might be one of the earliest biomarkers and could thus be used in therapeutic screens or as a diagnostic tool.

## Regulation of axonal function by FUS

Some studies have implicated FUS in the regulation of neuronal pre-synaptic function with disease-associated FUS mutants impairing its regulatory role (Sasayama et al., 2012; Armstrong and Drapeau, 2013; Schoen et al., 2015). Errichelli et al. reported that circular RNA expression, which is involved in axon guidance, was affected in motor neurons of FUS KO mice (Errichelli et al., 2017). Axonal transport defects have been reported for ALS/FTLD-associated mutations of FUS (Baldwin et al., 2016; Chen et al., 2016). Moreover, Guo et al. found that axonal transport was affected by disease-associated mutations of FUS in human iPSC-derived motor neurons (Guo et al., 2017). Since axonal transport defects appear in mice carrying mutations in SOD1 that cause ALS and in Drosophila carrying mutations in TDP-43 and C9orf72 (Williamson and Cleveland, 1999; Baldwin et al., 2016), further investigation to clarify the common downstream pathomechanism is necessary.

## Liquid-phase transition of FUS and its pathological and physiological functions

Recent studies have unveiled a novel protein property of FUS, liquid-liquid phase transitions that lead to the formation of various proteinaceous membrane-less organelles. It has been demonstrated that FUS undergoes a liquid–liquid phase separation before converting into the insoluble form of the protein, a process that is promoted by mutations, phosphorylation, or the presence of RNA (Murakami et al., 2015; Patel et al., 2015; Chong and Forman-Kay, 2016; Monahan et al., 2017). Similar to hnRNPA2, the low complexity domain (LCD) in the C-terminal region of FUS is responsible for the liquid–liquid phase separation (Xiang et al., 2015; Murray et al., 2017). Other RNA-binding proteins such as TDP-43, TIA1, TAF15, and dipeptide repeat proteins synthesized from mutant C9orf72 also contain LCDs (Boeynaems et al., 2017; Harrison and Shorter, 2017). Moreover, it has been reported that Tau also undergoes a liquid–liquid phase separation in solution with 4R-Tau more prone to form liquid droplets than 3R-Tau (Ambadipudi et al., 2017).

These findings strongly suggest a biochemical link between RNA-binding proteins and other amyloid-formable proteins including Tau and its association with RNA processing in neurodegenerative diseases. Since those findings were based on *in vitro* experiments, further investigation is necessary to clarify whether/how liquid-liquid phase transitions are associated with biological function, and whether transitions that occur in the cytoplasm of dendritic spines and/or the nucleus utilize the same or a different molecular process.

## Conclusions

**Figure 2:**
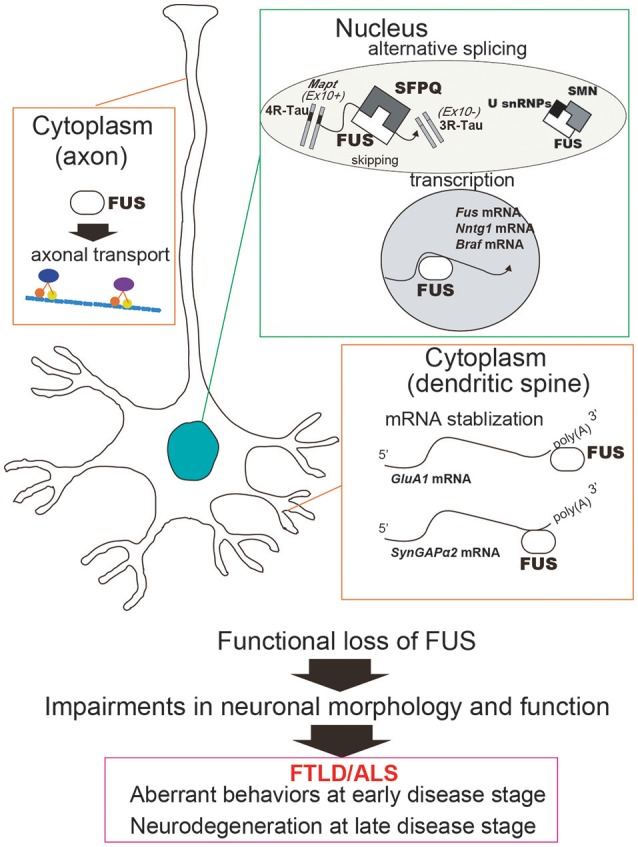
Functional loss of FUS in the nucleus and the cytoplasm can cause neuronal dysfunction and degeneration. In the nucleus, FUS regulates alternative splicing and transcription. For instance, exon 10 skipping of *MAPT*, which is regulated by FUS in complex with SFPQ, generates two isoforms of Tau protein, 3R-Tau and 4R-Tau. FUS also regulates transcription of a number of genes including *Ntng1, Braf1*, and *Fus* itself. On the other hand, cytoplasmic FUS stabilizes mRNAs involved in the dendritic spine, such as *GluA1* and *SynGAP*. Taken together, the functional impairments caused by FUS deficiency can affect neuronal function and morphology and subsequently lead to aberrant behaviors and neurodegeneration. In addition, FUS has also been implicated in the axon transport machinery, which is impaired by disease-associated mutations in FUS.

Accumulating *in vitro* and *in vivo* evidences indicate that FUS dysfunction might be involved in the pathomechanism of FTLD/ALS and other neurodegenerative diseases including tauopathies. FUS directly impacts RNA metabolism via alternative splicing, transcription, and mRNA stabilization, all of which can subsequently influence neuronal/synaptic functions and lead to impaired behaviors during the early disease stage and neurodegeneration at the late disease stage (Figure 2). Although this review focused on the loss of FUS function, FUS toxicity could affect RNA metabolism as well; for instance, overexpression of mutant FUS has been shown to disrupt target gene expression (Coady and Manley, 2015). It indicates that loss-of-function and/or gain-of-toxicity of FUS might influence RNA metabolism pathways and subsequent cellular phenomenon. Indeed, in neurons with simultaneous depletion of FUS and TAF15 the gene expression profiles were similar to that in ALS patient-derived neurons bearing the ALS mutation FUS^R521G^ (Kapeli et al., 2016). This is further supported by similar transcriptome profiles in TDP-43 Drosophila models following both loss and gain of FUS function (Vanden Broeck et al., 2013). Consequently, to determine the utility of FUS and its downstream pathways as early disease markers and/or therapeutic targets of FTLD/ALS, it is crucial that their functional properties be more precisely clarified.

## Author contributions

SI: Conception and design, manuscript writing, editing, and figure design. GS: Conception and design, manuscript writing, and editing.

### Conflict of interest statement

The authors declare that the research was conducted in the absence of any commercial or financial relationships that could be construed as a potential conflict of interest.
